# Pretreatment of Wheat Straw Lignocelluloses by Deep Eutectic Solvent for Lignin Extraction

**DOI:** 10.3390/molecules27227955

**Published:** 2022-11-17

**Authors:** Hongzhi Ma, Penglu Fu, Jihua Zhao, Xingxing Lin, Wenyu Wu, Ziqiang Yu, Changlei Xia, Qunhui Wang, Ming Gao, Jun Zhou

**Affiliations:** 1 Department of Environmental Science and Engineering, University of Science and Technology Beijing, Beijing 100083, China; 2 Beijing Key Laboratory of Resource-Oriented Treatment of Industrial Pollutants, Beijing 100083, China; 3 Jiangsu Co-Innovation Center of Efficient Processing and Utilization of Forest Resources, International Innovation Center for Forest Chemicals and Materials, College of Materials Science and Engineering, Nanjing Forestry University, Nanjing 210037, China; 4 College of Management Academician Workstation, Changsha Medical University, Changsha 410219, China

**Keywords:** wheat straw, pretreatment, deep eutectic solvent, lignin, cellulose

## Abstract

In order to increase the fractionation efficiency of the wheat straw, a deep eutectic solvent (DES) system consisting of chlorine/lactic acid was used in this study for wheat straw pretreatment. The outcomes exhibited that DES pretreatment significantly enhanced the capability to extract lignin, retain cellulose, and remove hemicellulose. The best condition for the pretreatment of wheat straw was 150 °C for 6 h. The process retained most cellulose in the pretreated biomass (49.94–73.60%), and the enzymatic digestibility of the pretreatment residue reached 89.98%. Further characterization of lignin showed that the high yield (81.54%) and the high purity (91.33%) resulted from the ether bond cleavage in lignin and the connection between hemicellulose and lignin. As for application, the enzymatic hydrolysis of the best condition reached 89.98%, and the lignin also had suitable stability. The investigation exhibited that DES pretreatment has the potential to realize an efficient fractionation of lignocellulosic biomass into high-applicability cellulose and lignin of high-quality.

## 1. Introduction

Fossil energy depletion and growing environmental problems are impeding sustainable socio-economic growth, and there has recently been a strong interest in clean and renewable energy [1,2]. As a renewable carbon source featuring an annual output of approximately 200 billion tons, lignocellulose has attracted much attention for its capacity for producing biofuels and high-value-added chemicals [3,4]. Nonetheless, resulting from its compact structure composed of three major polymers, lignin (15–30 wt%), hemicellulose (20–25 wt%), and cellulose (30–50 wt%), the high resistance of this biological structure is typical [5,6]. Straw is one of the most common lignocellulosic wastes that are produced by crops during their agricultural cultivation [7]. For example, 8–13 million tons of cereal straw is generated every year in Germany, whereas about 12.2 million tons of straw was produced in 2011 in the UK from cereals and oilseed. Globally, wheat straw is the most important by-product of wheat processing produced in larger quantities [8,9]. In order to make effective use of the three parts of the wheat straw, the applicable pretreatment step is often necessary; for instance, the effectiveness of saccharification is greatly dependent on removing stubborn structures comprising lignin and hemicellulose [10,11].

Many fractionation technologies are utilized for overcoming the recalcitrance of biomass and opening up the complex structure of lignocellulose, such as ionic liquids (ILs) pretreatment [12,13], alkali treatment [14,15], or physical pretreatment [16]. Mechanical milling with electricity as the main power source faces the obstacles of low saccharification efficiency and high energy consumption [17]. As an efficient pretreatment approach, alkaline hydrolysis is broadly adopted in the deconstruction of biomass. Nonetheless, the major problem has restricted industrial application, which is attributed to the difficulty of separating lignin and hemicellulose [18,19]. The application of ionic liquids (ILs) in biomass pretreatment is increasingly identified as an effective and environmentally friendly method. However, ILs have a fundamental disadvantage in terms of high cost. In addition, its generalization and industrial application are also difficult due to the complexity of the synthesis process [20]. Given the shortcomings of current pretreatment technologies, the development of sustainable and skilled methods to improve biomass processing is an ongoing impetus.

Recently, deep eutectic solvent (DES), a co-crystal blend of hydrogen bond acceptor (HBA) and hydrogen bond donor (HBD), has been developed as an emerging method for deconstructing the cell walls of grass and wood under proper conditions. So far, DESs have attracted more attention in biomass fractionation due to their low cost, ease of synthesis, and biodegradability. In addition, other performance issues, such as environmental protection and low toxicity, can also be addressed [21]. In the field of biomass fractionation, DES pretreatment efficiently extracted hemicellulose and lignin while keeping the cellulose intact with no serious degradation, causing obvious changes in cell wall polymer characteristics and greater accessibility to enhance cellulase hydrolysis [22,23]. Typically, choline chloride-based DESs are identified by most investigators [24,25]. Many biomass-derived carboxylic acids, such as oxalic acid (OA), lactic acid (LA), and formic acid (FA), are utilized as HBDs in DES syntheses. The delignification efficiency of DES in this category varies depending on the type of carboxylic acid and pretreatment conditions. Zhang and colleagues [26] reported the highest delignification of 98.5% using corncob as feedstock and CHCl:OA as a pretreatment solvent. Chloride/lactic acid also exhibited excellent properties in removing lignin from grass and woody biomass and could remove lignin up to 93.1% [26]. The molar ratio of carboxylic acid and CHCl impacts the extraction efficiency of lignin as well. For instance, the extraction of lignin rose from 64.7% to 93.1% by increasing the molar ratio of lactic acid to chloride from 2 to 15 [26]. This increase in lignin extraction can result from more active protons provided by LA, facilitating the proton-catalyzed cleavage of different bonds in biomass (e.g., lignin prime-polysaccharide bonds, glycosidic bonds in polysaccharides, ether bonds in lignin). Overall, carboxylic acid-based DES pretreatment is efficient in removing lignin but requires longer pretreatment times, ranging between 3 and 24 h, at temperatures between 60 and 150 °C. From an economical and practical point of view, the preprocessing performance needs to be improved. Therefore, the feasibility of DES recycling for corresponding cellulose saccharification and lignocellulose deconstruction was intensively investigated. In addition, lignin extracted from the new DES would be an ideal raw material for carbon materials and fuels. This provided a more comprehensive understanding of the properties of lignin and more opportunities for its utilization.

A DES system consisting of lactic acid/choline chloride (LA/CHCl) is presented for deconstructing wheat straw under eight different temperature and time conditions. The structural alterations and enzymatic hydrolyses efficiency of the acquired cellulosic materials were discussed. In addition, lignin thermochemistry was systematically studied. It is generally believed that an in-depth understanding of the deconstruction process of chloride/lactic acid will contribute to the industrialization of effective pretreatment strategies in current biorefinery chemistry. Overall, the investigation integrated physical and chemical approaches toward complete lignocellulosic biomass utilization, which was proved to be a green course. Effective fractionation of lignocellulosic biomass was achieved in this study.

## 2. Results and Discussion

**Figure 1:**
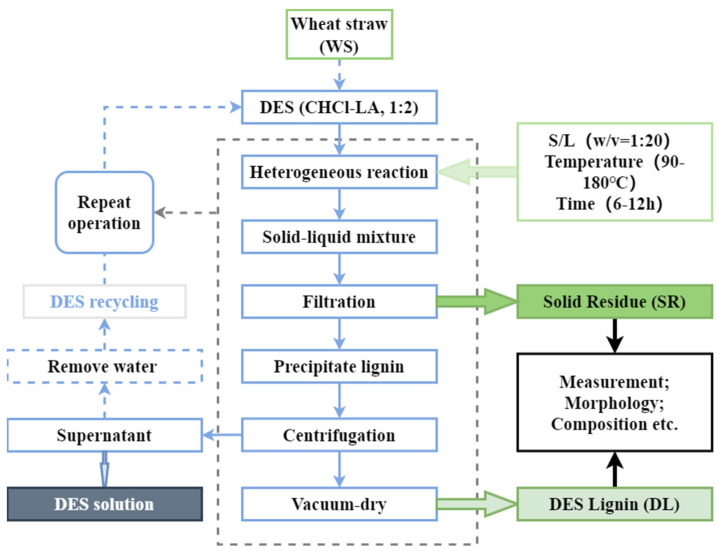
Flow chart for lignin extracted by DES.

The flow chart for the study of lignin extracted by DES was drawn in Figure 1. In the investigation, wheat straw was pretreated by utilizing chloride/lactic acid DES solution 1:20 (*w:v*) and at various temperatures (90 °C, 120 °C, 150 °C, and 180 °C) for 6 h and 12 h. Figure 1 schematically showed that DES fractionated biomass into three main parts: (1) pretreated wheat straw, which was high in cellulose (SR); (2) high-purity DES lignin (DL); (3) supernatant including hemicellulose and other components (DES solution). After an entire pretreatment course, the efficacy of the DES system solution decreased to a certain extent. After the steps of lignin precipitation and solid-liquid separation, the solution system containing DES dissolved matter used DES, ethanol, and water and was treated with a rotary evaporator to remove ethanol and water in the system.

During the entire pretreatment course, DES mainly acted on hemicellulose and lignin. Hence, in order to study the effect of reaction temperature and time on the pretreatment process, the reaction degree was curbed by modulating the reaction temperature and time. The removal rate of lignin was controlled and hemicellulose by DES, and adjust the ultimate product components. In addition, the potential utilization value of the DES-pretreated lignocellulosic biomass could be explored through the characterization and analysis of the components obtained after pretreatment.

### 2.1. Recovered Solids after Pretreatment of Wheat Straw by DES

**Figure 2:**
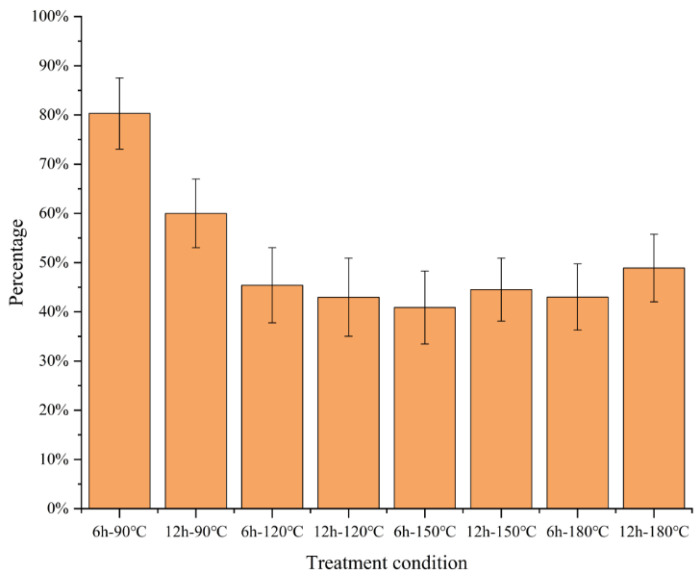
Recovered wheat straw solid residues obtained from various DES pretreatment conditions.

The wheat straw was pretreated according to the Section 3.2 and Section 3.3. After fractionating each component, each group of pretreated wheat straw was collected to recover solid residues (SR), the rate of solid recovery was the ratio of the solid residues of pretreated wheat straw to the original wheat straw, and it was calculated in Figure 2. After the ChCl-LA-based DES treatment, wheat straw was divided into three fractions: solute in DES, DES lignin, and solid residues. SR made up all insoluble biomass remaining after DES pretreatment. A high SR percentage means fewer dissolved solids after DES pretreatment. In this study, the pretreatment effect of wheat straw could be indicated clearly by SR.

The solid recovery rate of wheat straw decreased first and then increased, aligning with the investigation results of Lou [21] and Tan [27]. In the process of the pretreatment conditions, the fractionation effect was more obvious. A large amount of hemicellulose and lignin was extracted into the DES system, and most of the cellulose was retained until the fractionation reached a certain condition (150 °C and 6 h in this experiment). The solid recovery reached the lowest value of 40.88%, and then the solid recovery increased slightly.

At four temperatures (90 °C, 120 °C, 150 °C, and 180 °C) and two various reaction times (6, 12 h), how reaction time and treatment temperature affect the extraction of lignin through DES treatment was explored. At 90 °C, after DES treatment for as high as 6 h, only a small fraction of the straw was dissolved. When the treatment temperature arrived at 120 °C (45.40%) and 150 °C (40.88%), the SR yield began to decrease significantly. Notably, raising the treatment time to 180 °C did not decrease the SR further but increased it (44.53%).

In addition, except for the two groups of 90 °C and 6 h and 120 °C and 6 h, the temperature change of the pretreatment had no obvious impact on the solid recovery. The obvious drop in the recovery rate was due to the extraction of a considerable amount of hemicellulose and lignin, which was also confirmed in the study of Lou et al. [28]. The pretreatment temperature was more effective on the pretreatment effect of wheat straw.

The outcomes implied that temperature was an important consideration for ChCl-LA-based DES treatment of wheat straw, and there were optimal treatment conditions that maximized the lignin extraction rate.

### 2.2. Yield and Composition of Pretreated Wheat Straw

**Figure 3:**
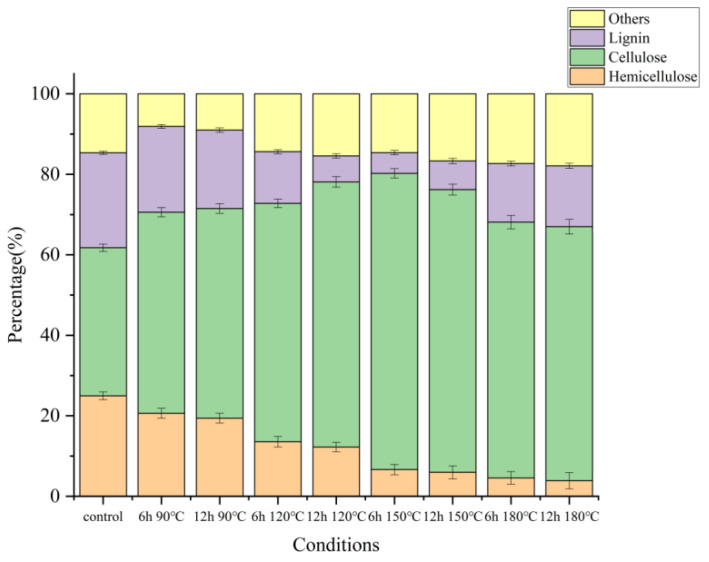
Chemical composition of wheat straw solid residues obtained from various DES pretreatment conditions.

To understand how DES pretreatment affected wheat straw structural changes and enzymatic digestibility, changes in cellulose, hemicellulose, and lignin were quantified, and the results were shown in Figure 3 (specific data were shown in Table S1 in the supporting material). It is essential to determine the chemical composition of the recovered solids accurately. The chemical compositions of initial wheat straw and DES-pretreated solid residues are shown in Figure 3. The initial wheat straw consisted of cellulose (36.77% ± 0.19%), hemicellulose (24.98% ± 0.16%), and lignin (23.59% ± 0.12%).

The chemical composition of the initial wheat straw changed obviously after DES treatment. Notably, the hemicellulose percentage dropped as the treatment temperature increased. Compared with the original hemicellulose content of 24.98%, the samples pretreated from 150 °C to 180 °C showed significantly lower hemicellulose content, ranging from 3.87% to 6.64%, which meant that high temperature was conducive to the dissolution of hemicellulose and chemical degradation. At the same time, a large amount of lignin in the wheat straw was extracted into the DES system, and the lignin content was reduced (from 23.59% in the original sample to 5.14% in the lowest group). This proved the effectiveness of lignin extraction under the given pretreatment conditions. The extraction of lignin and hemicellulose was caused by the fact that DES pretreatment could destroy carbohydrate complexes in the structure of wheat straw to remove hemicellulose, and at the same time, the ether bonds in lignin were disintegrated to enter the DES system.

The solid recovery of 80.31% and 60.00% were obtained under the pretreatment condition of 90 °C and 6 h and 90 °C and 12 h, respectively, in which the hemicellulose content was 20.63% and 19.42%, respectively. This implied that treatment temperature had little effect on the effect of pretreatment. Under pretreatment conditions of 150 °C and 6 h, the extraction rate and the purity of lignin reached the highest at 81.54% and 91.33%, respectively. However, the solid recovery rate of 150 °C and 6 h was the lowest at 40.88%. However, further raising the pretreatment temperature to 180 °C did not promote the dissolution effect, which was consistent with the changing trend of solids recovery.

**Figure 4:**
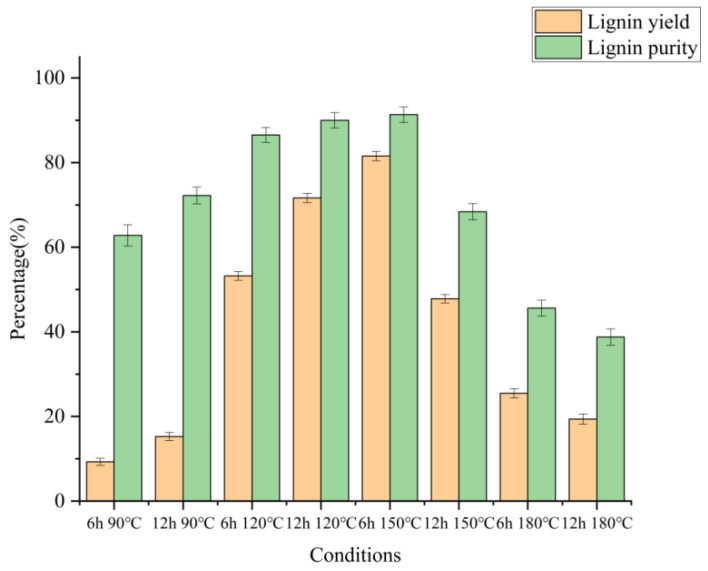
Extracted DES lignin yield of wheat straw obtained from various DES pretreatment conditions.

To clarify the extraction effect of lignin, the relevant components and contents of lignin were also analyzed in Figure 4 (specific data were shown in Table S2 in the supporting material). The lignin output varied from 15.26% to 81.54%, corresponding to continuous extraction by increasing the temperature from 90 °C to 180 °C, with the highest yield of 81.54%. The purity of lignin could reach 91.33%, which was higher than the 90.8% purity of regenerated lignin extracted from wheat straw and the highest purity of 88.6% of lignin under the same conditions [21,29]. This indicated that lignin extraction by chloride/lactic acid had a higher purity, which was beneficial to the decomposition and further utilization of the wheat straw structure.

The lignin purity was affected by the reaction time and temperature of the DES treatment. Increasing the treatment temperature from 90 °C and 6 h to 150 °C and 6 h results in significant improvements in both the lignin yield and lignin purity. However, increasing the treatment time further could not improve the lignin yield and purity. Lignin macromolecule contains a huge number of functional groups, such as aliphatic and phenolic hydroxyl groups. In the wheat straw cell wall, lignin is also linked to hemicellulose via various alkyl/aryl ether bonds to form the lignin-carbohydrate complexes [30]. The increase in temperature made the protons more active, causing full β-O-4 bond cleavage in lignin. Meanwhile, with the increase in temperature, DES cleaved the bonds between hemicellulose and lignin and the ether bonds in lignin, which might be an important factor in promoting lignin extraction [21]. As the pretreatment became more stringent, the cellulose structure in the system began to collapse and gradually converted into other chemicals (such as sugar chaff). On the other hand, the lignin molecules were treated so seriously that they were harder to extract, and some lignin molecules were adhered to the surface of wheat straw, so the lignin yield and lignin purity decreased.

Figure 3 and Figure 4 showed that under the condition of 150 °C for 6 h, the cellulose (73.60%) in wheat straw was preserved to the greatest extent in this study. Most of the hemicellulose was removed (6.64% remained), and most of the lignin was extracted from the extracted lignin. The yield and purity of lignin were 81.54% and 91.33%, respectively. It could be judged that 150 °C and 6 h was the best pretreatment condition for the study, which was consistent with the change in solid recovery rate.

### 2.3. Microstructure Analysis of Pretreated Wheat Straw

**Figure 5:**
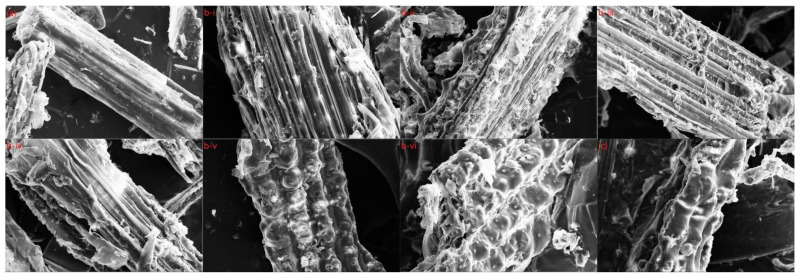
SEM picture of WS: (**a**) unpretreated; (**b**) (**b-i**) (90 °C for 6 h), (**b-ii**) (90 °C for 12 h), (**b-iii**) (120 °C for 6 h), (**b-iv**) (120 °C for 12 h), (**b-v**) (150 °C for 6 h), and (**b-vi**) (150 °C for 12 h); (**c**) 180 °C.

For a better understanding of the structural alterations of wheat straw during pretreatment, including fiber destruction and increasing porous structure, the morphologies of the initial and pretreated specimens were investigated by SEM (scanning electron microscopy). The SEM images were divided into three groups: Figure 5a was the unpretreated wheat straw, Figure 5b showed the wheat straw treated at different temperatures and times, b-i (90 °C for 6 h), b-ii (90 °C for 12 h), b-iii (120 °C for 6 h), b-iv (120 °C for 12 h), b-v (150 °C for 6 h), and b-vi (150 °C for 12 h), and Figure 5c showed the wheat straw pretreated at 180 °C.

As shown in Figure 5a, natural biomass had a complete and smooth surface featuring an ordered fibrous structure. At the same time, the non-porous and rigid structure significantly prevented enzyme molecules from going into the wheat straw, so the cellulose accessibility was restricted. This was in contrast to Figure 5b,c, in which DES-pretreated wheat straw exhibited fiber separation and slight cracks at elevated temperatures. Furthermore, resulting from the removal of lignin and hemicellulose, the dense fiber bundles became loose and disorganized, which facilitated enzyme hydrolyses and penetration. Therefore, during the enzymatic hydrolysis, the cellulase obtained a suitable environment, and the enzymatic digestion efficiency of the cellulase was significantly improved. The more lignin and xylan removed, the greater the exposure of surface area and disordered fibrils, thus, improving cellulose accessibility [31,32,33]. Moreover, the resulting solid residue surface was not as smooth as anticipated since the residue after DES pretreatment still contained some non-fibrous components.

If the pretreatment temperature was constant, as the pretreatment temperature increased, the pretreatment effect of wheat straw increased, and the removal of hemicellulose and lignin increased correspondingly, which was consistent with the specific data of recovered solids anastomosis (Table S1). However, when the pretreatment conditions reached a certain level, namely Table S1, the morphology of the recovered solids at 180 °C had been severely damaged, and at the same time, the solid recovery rate at 180 °C began to decrease slightly, the overall pretreatment effect also deteriorated. In general, as the severity of pretreatment gradually deepened, the pretreatment effect also increased steadily, but when it reached a certain value (150 °C and 6 h in this experiment), the pretreatment effect no longer continued to improve and showed a downward trend. Combined with the solid recovery rate, the group of 150 °C and 6 h was the group with the best pretreatment effect.

### 2.4. Microstructure Analysis of Pretreated Wheat Straw

**Figure 6:**
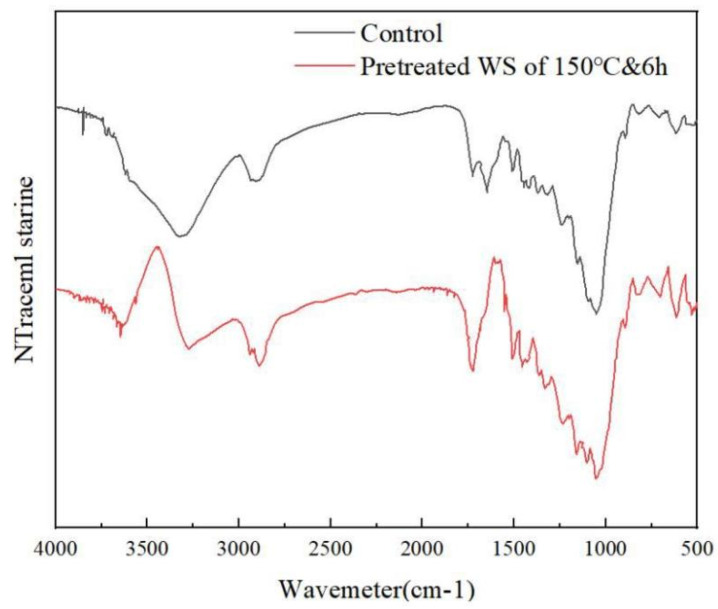
FTIR spectra of untreated and DES-pretreated wheat straw.

FTIR was performed to identify changes made on wheat straw after 150 °C and 6 h pretreatment. It is shown in Figure 6. Resulting from changes in β-glycosidic bonds between hemicellulose, cellulose, and glucose polymers, the C-O-C stretching of b-glycosidic bonds between sugar units in hemicellulose and cellulose exhibited uptake at 895, 1031, and 1159 cm^−1^ [34], which might be interpreted based on a large hemicellulose removal proportion [35]. The flattening of the peak at 1735 cm^−1^ indicated that in the hemicellulose carbonyl ester, the C=O acetyl group was obviously destroyed [34], and the absorption peak here was mainly from the carbonyl group in the hemicellulose (partly from the cellulose). The DES-pretreated samples exhibited significantly lower uptake compared to the untreated samples, indicating that a large amount of hemicellulose (from 24.98% to 6.64%) was removed during the pretreatment process, an observation consistent with the component content analysis results in Figure 3 [36]. The signal at 1363 cm^−1^ was assigned to C-H stretching, associated with amorphous and crystalline cellulose, respectively. At 1435 cm^−1^, the small peak reduction corresponded to the C-C stretching vibration of lignin ring aromatics [36]. At 2900 cm^−1^, the peak implied that alkane functional groups were discovered in the C-H stretching vibration, which indicated that a large amount of lignin was extracted into the DES system during the pretreatment process, thus reducing the lignin content in the pretreated wheat straw. As for the large peak band between 3000 and 3500 cm^−1^, it was attributed to the change of much hydroxyl-OH during the pretreatment process, where the vibration was extremely obvious, indicating that DES extracted many aromatic rings in wheat straw, the treated wheat straw polarity was weakened, and the binding ability with -OH was greatly reduced. Combined with the changes in component content (Figure 3), the FTIR spectrum proved that the pretreatment process could achieve the purpose of biomass fractionation of wheat straw.

### 2.5. Enzymatic Saccharification

**Figure 7:**
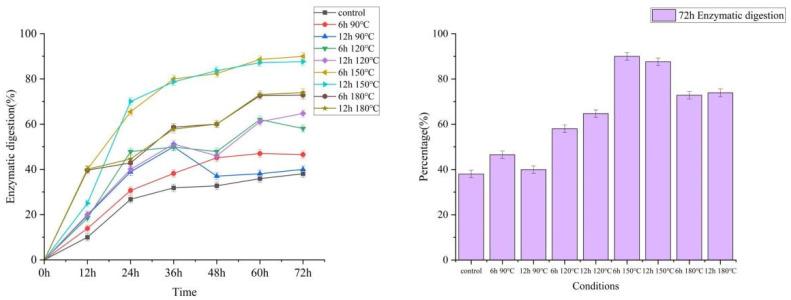
Enzymatic digestion of pretreated WS residues by DES.

To study the potential utilization value of pretreated wheat straw, further enzymatic digestibility assessments were performed on all pretreated solid residues. Figure 7 showed that when the pretreatment time was 6 h, enzymatic hydrolysis digestibility increased significantly as treatment temperature increased. The 72 h enzymatic digestion rate of 90 °C, 120 °C, 150 °C, and 180 °C were 46.56%, 58.06%, 89.98%, and 72.81%, respectively; when the pretreatment time was 12 h, the 72 h enzymatic digestion rates of 90 °C, 120 °C, 150 °C, and 180 °C were 39.97%, 64.72%, 87.63%, and 73.90%, respectively. This is similar to the study of Xie et al. [37]. However, it is worth noting that if the temperature increased from 150 °C to 180 °C, the enzymatic digestibility decreased from 89.98% to 72.81%, and the same trend was for the 12 h group, from 87.63% to 73.90%. If the temperature increased from 90 °C to 150 °C, the pretreatment would break the chemical bonds between hemicellulose, cellulose, and lignin, thereby significantly improving the enzymatic digestion rate. When the temperature was too high, that was, 180 °C, the pretreatment would cause more serious damage to the connection between lignin, cellulose, and hemicellulose, and this damage would make the broken lignin fragments cover the fibers again. Similar speculations and findings have been proposed by some researchers, such as Liu et al. [38], Isci et al. [39], and Chen et al. [40]. In fact, according to the SEM, as the treatment temperature increased, Cracks and crevices slowly began to appear on the smooth surface of the wheat straw, and then the wheat straw gradually became loose and disorganized. When the pretreatment temperature reached 180 °C, the structure of the wheat straw collapsed seriously. The surface coverage was also reduced. The 72 h enzymatic digestion rate ranged from 46.56% at 90 °C and 6 h to 39.97% at 90 °C and 12 h, from 58.06% at 120 °C and 6 h to 64.72% at 12 °C and 12 h, and from 89.98% at 150 °C and 6 h to 87.63% of 150 °C and 12 h, from 72.81% at 180 °C and 6 h to 73.90% at 180 °C and 12 h, there were two points worth noting here: firstly, the pretreatment effect was much worse than that of the pretreatment temperature; secondly, in the two groups of 90 °C and 180 °C, the pretreatment effect decreased with the increase in the pretreatment time. It proved that the intensity of pretreatment time was not as large as that of pretreatment temperature, the increase in pretreatment time would have a negative effect. When the pretreatment temperature was too high, the solid and liquid mixed in the slurry, the large lignin fragments covered the cellulose surface again for a long time, which reduced the digestibility during the enzymatic hydrolysis process. Combined with the effects of pretreatment temperature and time, the optimal pretreatment group was 150 °C and 6 h.

### 2.6. Thermal Analyses of Lignin

**Figure 8:**
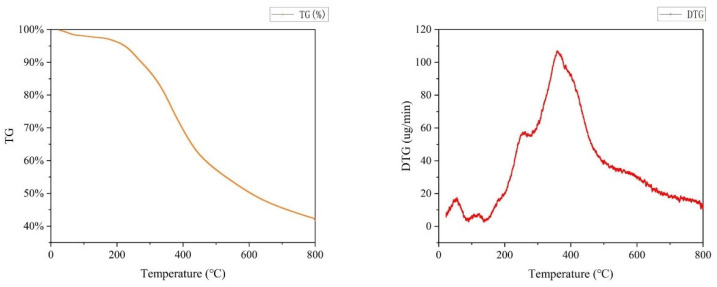
TG and DTG curve of lignin.

Thermogravimetric (TG) analyses verified the percent weight loss and thermal stability of lignin specimens. It was an important characteristic of lignin and had practical implications, especially in the field of application. Therefore, the TG plots of lignin separated at 150 °C and 6 h were carried out at a heating rate of 10 °C/min, the onset temperature is 20 °C, and the outcomes are shown in Figure 8.

First, rapid weight loss occurred at 25–100 °C, which resulted from water molecule capture through the hydrophilic groups of lignin [41], and the removal of volatiles [42]. After that, a mild stage at 100–200 °C was associated with the cleavage of weak C-O bonds in β-O-4’ bonds and dehydration of hydroxyl groups [42]. When the temperature reached 200–450 °C, inter-unit bonds such as aryl ether bonds started pyrolyzing, and it was discovered that the slope of the TG curve was the largest here, which was due to the fastest decomposition rate at this stage due to lignin oxidation. There were two main stages of this decomposition process, which typically occurred around 200 °C and 350 °C, respectively. The weight loss (WL) around 200 °C was mainly attributed to the degradation of small molecular lignin and lignin side chains [43]. The onset temperature of the first phase was regarded as the main parameter for thermal material application in plastics [44]. The second WL of DES lignin at 350 °C majorly resulted from the serious cleavage of the internal bonds of lignin after further heating, causing the production of small-molecule volatiles [45]. Moreover, it was evident that after 450 °C, the thermal mass of each lignin specimen decreased mildly. This might result from the lignin condensation reaction at high temperatures, forming macro-molecular refractory products [1]. Robust aromatic rings and C-C bonds decomposed mildly above 500 °C. Furthermore, the DES lignin obtained in this study exhibited a rather high coke residue resulting from forming fused C-C bonds [46]. About 41.5% of the coke was obtained when the temperature reached 800 °C. The solid char residue sulfate lignin showed 47.8% residual coke, and the DES-extracted lignin (DEL) featured only 33.4% residue, indicating a more concentrated C-C bond in the sulfate lignin in comparison with the DEL [47]. The lower quantity of C-C bonds in the EL might contribute to its easier value-added to aromatics [47,48], which implies it is greater potential for application in the production of chemicals. DTG analysis was also performed, and the DTG curve was related to water removal at low temperatures (50 to 150 °C) [48]. Furthermore, a sharp maximum was registered at 300 °C, caused by β-O-4 bond cleavage [49]. The shoulder at larger temperature (450–550 °C) corresponds to C-C bond cleavage in lignin [49]. The peak around 700 °C corresponds to the forming of condensed lignin structures through polymerization reactions/condensation that occur at larger temperatures [47,48].

In summary, the DES fractionation of lignin has great potential for pyrolysis to produce coke and carbon materials.

## 3. Materials and Methods

### 3.1. Materials

Wheat straw came from Lianyungang City, Jiangsu Province, China. The wheat straw specimens were ground with a grinder to a particle size of 60–80 mesh, consisting of 36.77% cellulose, 24.98% hemicellulose, 23.59% lignin, and 15.66% other components, and then vacuum dried at 105 °C for more than 48 h. Cellulase with 150 FPU/mL pilocytic activity was provided by Novozymes, China. LA and CHCl came from Aladdin Industrial Corporation. All other chemicals were of analytical grade and were employed without being further purified.

### 3.2. DES Preparation

DES mixture was carried out with choline chloride and LA in a molar ratio of 1:2. Specifically, 139.63 g CHCl and 180.16 g LA were mixed and heated for two hours at 80 °C. After obtaining a clear mixture free of solid particles, the DES was cooled in a desiccator to prevent moisture absorption [29].

### 3.3. DES Pretreat Wheat Straw

Pretreatment was performed in a 100 mL round-bottom flask containing 40 mL of DES and 2 g of wheat straw. The blend was heated to the target temperature (90 °C, 120 °C, 150 °C, 180 °C) and preserved at the target temperature for 6 h and 12 h under constant magnetic stirring. After the reaction, DES liquid and solid fractions were vacuum filtered with 50% ethanol/water (300 mL, *v/v*) until the filtrate was neutral. The mixture of DES and residues was evaporated to remove ethanol using a rotary evaporator at 65 °C. Meantime, after standing for about 2 h, a precipitated solid was obtained, which was defined to be regenerated lignin. The lignin was collected through vacuum filtration using a 0.45 μm membrane and washed with deionized water. The method was adjusted based on the study of Lou et al. [21].

Lignin was precipitated, then the further liquid mixture was concentrated by adopting a rotary evaporator for two hours at 60 °C to remove the remaining water in the DES system.

### 3.4. Enzymatic Hydrolyses

In a 25 mL round-bottom flask, by adopting 300 mg biomass (dry weight), enzymatic hydrolysis of untreated and pretreated wheat straw was performed at 5% (*w/v*) biomass loading. A total of 50 mM sodium acetate buffer was added to maintain a proper enzyme environment (PH = 4.8). The solid residue was loaded with 15 FPU/g for cellulose. Samples were withdrawn at 72 h, 60 h, 48 h, 36 h, 24 h, 12 h, and 6 h. When it reached the desired enzymatic hydrolysis time, the reaction was stopped by sealing 0.2 mL of the supernatant for five minutes in a boiling water bath. Then, the saccharification kinetics was monitored by applying high-performance liquid chromatography (HPLC-20AT, Shimadzu, Tokyo, Japan) with a refractive index detector (RID-10A, Shimadzu, Japan); the column was a Shodex SH1011 column (Showa Denko K.K., Tokyo, Japan). The enzymatic hydrolysis residue was dried and stored for further use. Three replicate experiments were performed for each enzymatic hydrolysis step. Enzymatic digestibility was defined as the amount of glucose produced by enzymatic hydrolysis to the glucan content in wheat straw [37].

### 3.5. Characteristics of the Pretreated Wheat Straw

Based on the National Renewable Energy Laboratory (NREL) standard [50], the compositions of the raw and pretreated wheat straw were determined. All assays were performed in triplicate and presented as average values. Monosaccharide quantification was performed at 45 °C by HPLC, consisting of a refractive index detector by employing a column eluted with deionized water at 60 °C. The flow rate was 0.6 mL·min^−1^. Additional support for the extent of wheat straw after pretreatment was determined through FTIR. A Nicolet-6700 spectrometer (Thermo Scientific, Waltham, MA, USA) was used to observe FTIR through a spectrophotometer in the wavelength scope of 4000–500 cm^−1^. The morphology of the cellulose residue was explored by adopting a scanning electron microscope (SEM) (ZEISS, Jena, Germany, GeminiSEM300). The accelerating voltage was 10 kV.

### 3.6. Characterization of Lignin

The purity of lignin was calculated based on the total amount of acid-soluble lignin (ASL) and acid-insoluble lignin (AIL) as a percentage of the total weight of the recovered lignin determined according to standard procedures [29]. Similarly, the average values were used for analysis. Thermogravimetric analyses (TG) of the specimens were tested on a thermal analyzer (TA Instruments Thermogravimetric Analyzer (TGA Q500), Waltham, MA, USA) system. Heat the specimens from 20 °C to 800 °C at 10 °C·min^−1^ under a nitrogen atmosphere (50 mL·min^−1^).

## 4. Conclusions

This study presented a comprehensive understanding of the pretreatment of lignocellulose using a low-cost chloride/lactic acid system. The solution exhibited considerable selective extraction of hemicellulose and lignin while retaining substantial amounts of cellulose. The enzymatic saccharification was the highest after pretreatment at 150 °C for 6 h, reaching 89.98%, indicating that the pretreated wheat straw has a high utilization rate. DES pretreatment realized effective lignocellulosic biomass fractionation into high-quality lignin and highly adaptable cellulose.

## Data Availability

The data presented in this study are available in the article.

## Supplementary Materials

The following supporting information can be downloaded at: https://www.mdpi.com/article/10.3390/molecules27227955/s1, Table S1: Impact of various pre-treatment conditions on wheat straw chemical composition; Table S2: Yield and composition of lignin under different conditions.

## Sample Availability

Samples of the compounds are available from the authors.

## References

[1] Liu X., Li T., Wu S., Ma H., Yin Y. (2020). Structural characterization and comparison of enzymatic and deep eutectic solvents isolated lignin from various green processes: Toward lignin valorization. Bioresour. Technol..

[2] Sheng Y., Lam S.S., Wu Y., Ge S., Wu J., Cai L., Huang Z., Le Q.V., Sonne C., Xia C. (2021). Enzymatic conversion of pretreated lignocellulosic biomass: A review on influence of structural changes of lignin. Bioresour. Technol..

[3] Lynd L.R. (2017). The grand challenge of cellulosic biofuels. Nat. Biotechnol..

[4] Taherzadeh M.J., Karimi K. (2008). Pretreatment of lignocellulosic wastes to improve ethanol and biogas production: A review. Int. J. Mol. Sci..

[5] Mathew A.K., Parameshwaran B., Sukumaran R.K., Pandey A. (2016). An evaluation of dilute acid and ammonia fiber explosion pretreatment for cellulosic ethanol production. Bioresour. Technol..

[6] Mosier N., Wyman C., Dale B., Elander R., Lee Y.Y., Holtzapple M., Ladisch M. (2005). Features of promising technologies for pretreatment of lignocellulosic biomass. Bioresour. Technol..

[7] Yu Z., Ma H., den Boer E., Wu W., Wang Q., Gao M., Vo D.-V.N., Guo M., Xia C. (2022). Effect of microwave/hydrothermal combined ionic liquid pretreatment on straw: Rumen anaerobic fermentation and enzyme hydrolysis. Environ. Res..

[8] Tufail T., Saeed F., Afzaal M., Ain H.B.U., Gilani S.A., Hussain M., Anjum F.M. (2021). Wheat straw: A natural remedy against different maladies. Food Sci. Nutr..

[9] Lai C., Tu M., Xia C., Shi Z., Sun S., Yong Q., Yu S. (2017). Lignin Alkylation Enhances Enzymatic Hydrolysis of Lignocellulosic Biomass. Energy Fuels.

[10] Abaide E.R., Ugalde G., Di Luccio M., Moreira R., Tres M.V., Zabot G.L., Mazutti M.A. (2019). Obtaining fermentable sugars and bioproducts from rice husks by subcritical water hydrolysis in a semi-continuous mode. Bioresour. Technol..

[11] Sheng Y., Liu M., Xia C., Song J., Ge S., Cai L., Lam S.S., Sonne C. (2021). Using nucleophilic naphthol derivatives to suppress biomass lignin repolymerization in fermentable sugar production. Chem. Eng. J..

[12] Mamilla J.L.K., Novak U., Grilc M., Likozar B. (2019). Natural deep eutectic solvents (DES) for fractionation of waste lignocellulosic biomass and its cascade conversion to value-added bio-based chemicals. Biomass Bioenergy.

[13] Grilc M., Likozar B., Levec J. (2015). Kinetic model of homogeneous lignocellulosic biomass solvolysis in glycerol and imidazolium-based ionic liquids with subsequent heterogeneous hydrodeoxygenation over NiMo/Al_2_O_3_ catalyst. Catal. Today.

[14] Sun S., Chen W., Tang J., Wang B., Cao X., Sun S., Sun R.C. (2016). Synergetic effect of dilute acid and alkali treatments on fractional application of rice straw. Biotechnol. Biofuels.

[15] Yang J., Tu M., Xia C., Keller B., Huang Y., Sun F.F. (2019). Effect of fenton pretreatment on C1 and C6 oxidation of cellulose and its enzymatic hydrolyzability. ACS Sustain. Chem. Eng..

[16] Hoeger I.C., Nair S.S., Ragauskas A.J., Deng Y., Rojas O.J., Zhu J.Y. (2013). Mechanical deconstruction of lignocellulose cell walls and their enzymatic saccharification. Cellulose.

[17] Zhu W., Zhu J.Y., Gleisner R., Pan X.J. (2010). On energy consumption for size-reduction and yields from subsequent enzymatic saccharification of pretreated lodgepole pine. Bioresour. Technol..

[18] Liu X., Wei W., Wu S. (2019). Synergism of organic acid and deep eutectic solvents pretreatment for the co-production of oligosaccharides and enhancing enzymatic saccharification. Bioresour. Technol..

[19] Ge S., Wu Y., Peng W., Xia C., Mei C., Cai L., Shi S.Q., Sonne C., Lam S.S., Tsang Y.F. (2020). High-pressure CO_2_ hydrothermal pretreatment of peanut shells for enzymatic hydrolysis conversion into glucose. Chem. Eng. J..

[20] Yang T.-X., Zhao L.-Q., Wang J., Song G.-L., Liu H.-M., Cheng H., Yang Z. (2017). Improving Whole-Cell Biocatalysis by Addition of Deep Eutectic Solvents and Natural Deep Eutectic Solvents. ACS Sustain. Chem. Eng..

[21] Lou R., Ma R., Lin K.-T., Ahamed A., Zhang X. (2019). Facile Extraction of Wheat Straw by Deep Eutectic Solvent (DES) to Produce Lignin Nanoparticles. ACS Sustain. Chem. Eng..

[22] Alam A., Wang Y., Liu F., Kang H., Tang S.-W., Wang Y., Cai Q., Wang H., Peng H., Li Q. (2020). Modeling of optimal green liquor pretreatment for enhanced biomass saccharification and delignification by distinct alteration of wall polymer features and biomass porosity in *Miscanthus*. Renew. Energy.

[23] Hu M., Yu H., Li Y., Li A., Cai Q., Liu P., Tu Y., Wang Y., Hu R., Hao B. (2018). Distinct polymer extraction and cellulose DP reduction for complete cellulose hydrolysis under mild chemical pretreatments in sugarcane. Carbohydr. Polym..

[24] Kim K.H., Dutta T., Sun J., Simmons B., Singh S. (2018). Biomass pretreatment using deep eutectic solvents from lignin derived phenols. Green Chem..

[25] Yang R., Cao Q., Liang Y., Hong S., Xia C., Wu Y., Li J., Cai L., Sonne C., Le Q.V. (2020). High capacity oil absorbent wood prepared through eco-friendly deep eutectic solvent delignification. Chem. Eng. J..

[26] Zhang C.W., Xia S.Q., Ma P.S. (2016). Facile pretreatment of lignocellulosic biomass using deep eutectic solvents. Bioresour. Technol..

[27] Tan Y.T., Ngoh G.C., Chua A.S.M. (2019). Effect of functional groups in acid constituent of deep eutectic solvent for extraction of reactive lignin. Bioresour. Technol..

[28] Lou R., Zhang X. (2022). Evaluation of pretreatment effect on lignin extraction from wheat straw by deep eutectic solvent. Bioresour. Technol..

[29] Alvarez-Vasco C., Ma R., Quintero M., Guo M., Geleynse S., Ramasamy K.K., Wolcott M., Zhang X. (2016). Unique low-molecular-weight lignin with high purity extracted from wood by deep eutectic solvents (DES): A source of lignin for valorization. Green Chem..

[30] Huang C., Jiang X., Shen X., Hu J., Tang W., Wu X., Ragauskas A., Jameel H., Meng X., Yong Q. (2022). Lignin-enzyme interaction: A roadblock for efficient enzymatic hydrolysis of lignocellulosics. Renew. Sustain. Energy Rev..

[31] Ong V.Z., Wu T.Y., Chu K.K.L., Sun W.Y., Shak K.P.Y. (2021). A combined pretreatment with ultrasound-assisted alkaline solution and aqueous deep eutectic solvent for enhancing delignification and enzymatic hydrolysis from oil palm fronds. Ind. Crops Prod..

[32] Liu Y., Deak N., Wang Z., Yu H., Hameleers L., Jurak E., Deuss P.J., Barta K. (2021). Tunable and functional deep eutectic solvents for lignocellulose valorization. Nat. Commun..

[33] González-Rivera J., Mero A., Husanu E., Mezzetta A., Ferrari C., D’Andrea F., Bramanti E., Pomelli C.S., Guazzelli L. (2021). Combining acid-based deep eutectic solvents and microwave irradiation for improved chestnut shell waste valorization. Green Chem..

[34] Loow Y.L., Wu T.Y. (2018). Transformation of oil palm fronds into pentose sugars using copper (II) sulfate pentahydrate with the assistance of chemical additive. J. Environ. Manag..

[35] Lionetto F., Del Sole R., Cannoletta D., Vasapollo G., Maffezzoli A. (2012). Monitoring Wood Degradation during Weathering by Cellulose Crystallinity. Materials.

[36] Dungani R., Owolabi A.F., Saurabh C.K., Abdul Khalil H.P.S., Tahir P.M., Hazwan C.I.C.M., Ajijolakewu K.A., Masri M.M., Rosamah E., Aditiawati P. (2016). Preparation and Fundamental Characterization of Cellulose Nanocrystal from Oil Palm Fronds Biomass. J. Polym. Environ..

[37] Xie J., Chen J., Cheng Z., Zhu S., Xu J. (2021). Pretreatment of pine lignocelluloses by recyclable deep eutectic solvent for elevated enzymatic saccharification and lignin nanoparticles extraction. Carbohydr. Polym..

[38] Liu Y., Zheng J., Xiao J., He X., Zhang K., Yuan S., Peng Z., Chen Z., Lin X. (2019). Enhanced Enzymatic Hydrolysis and Lignin Extraction of Wheat Straw by Triethylbenzyl Ammonium Chloride/Lactic Acid-Based Deep Eutectic Solvent Pretreatment. ACS Omega.

[39] Isci A., Erdem G.M., Bagder Elmaci S., Sakiyan O., Lamp A., Kaltschmitt M. (2020). Effect of microwave-assisted deep eutectic solvent pretreatment on lignocellulosic structure and bioconversion of wheat straw. Cellulose.

[40] Chen J., Wang X., Zhang B., Yang Y., Song Y., Zhang F., Liu B., Zhou Y., Yi Y., Shan Y. (2021). Integrating enzymatic hydrolysis into subcritical water pretreatment optimization for bioethanol production from wheat straw. Sci. Total Environ..

[41] Chen Y., Jiang Y., Tian D., Hu J., He J., Yang G., Luo L., Xiao Y., Deng S., Deng O. (2020). Fabrication of spherical lignin nanoparticles using acid-catalyzed condensed lignins. Int. J. Biol. Macromol..

[42] Rashid T., Kait C.F., Regupathi I., Murugesan T. (2016). Dissolution of kraft lignin using Protic Ionic Liquids and characterization. Ind. Crops Prod..

[43] Lyu G., Wu Q., Li T., Jiang W., Ji X., Yang G. (2019). Thermochemical properties of lignin extracted from willow by deep eutectic solvents (DES). Cellulose.

[44] Song Y., Ji H., Shi X., Yang X., Zhang X. (2020). Successive organic solvent fractionation and structural characterization of lignin extracted from hybrid poplar by deep eutectic solvent for improving the homogeneity and isolating narrow fractions. Renew. Energy.

[45] Faravelli T., Frassoldati A., Migliavacca G., Ranzi E. (2010). Detailed kinetic modeling of the thermal degradation of lignins. Biomass Bioenergy.

[46] Chen Z., Bai X., A L., Zhang H., Wan C. (2020). Insights into Structural Changes of Lignin toward Tailored Properties during Deep Eutectic Solvent Pretreatment. ACS Sustain. Chem. Eng..

[47] Schorr D., Diouf P.N., Stevanovic T. (2014). Evaluation of industrial lignins for biocomposites production. Ind. Crops Prod..

[48] Moustaqim M.E., Kaihal A.E., Marouani M.E., Men-La-Yakhaf S., Taibi M., Sebbahi S., Hajjaji S.E., Kifani-Sahban F. (2018). Thermal and thermomechanical analyses of lignin. Sustain. Chem. Pharm..

[49] Kim J.-Y., Oh S., Hwang H., Kim U.-J., Choi J.W. (2013). Structural features and thermal degradation properties of various lignin macromolecules obtained from poplar wood (*Populus albaglandulosa*). Polym. Degrad. Stab..

[50] Sluiter A., Hames B., Ruiz R., Scarlata C., Sluiter J., Templeton D., Crocker D. (2008). Determination of Structural Carbohydrates and Lignin in Biomass.

