# Supplementary material for: Pretreatment of Wheat Straw Lignocelluloses by Deep Eutectic Solvent for Lignin Extraction
Source: Molecules. 2022 Nov 17;27(22):7955. doi: 10.3390/molecules27227955 (PMC9697946; doi:10.3390/molecules27227955)
Supplement: Supplementary file 1 [file molecules-27-07955-s001.zip › molecules-1981480-supplementary.pdf]

Table S1 Impact of various pre-treatment conditions on wheat straw chemical composition

Conditions	Recovery(%)	Hemicellulose(%)	Cellulose(%)	Lignin(%)		
				AIL*	ASL*	Total lignin
control	/	25.0±1.0	36.8±0.9	23.4±0.4	0.15	23.6±0.4
6h 90℃	80.3±0.9	20.6±1.2	49.9±1.1	21.2±0.5	0.13	21.3±0.5
12h 90℃	60.0±1.0	19.4±1.3	52.1±1.2	19.3±0.5	0.14	19.5±0.6
6h 120℃	45.4±1.1	13.6±1.3	59.2±1.1	12.7±0.5	0.12	12.9±0.5
12h 120℃	43.0±1.1	12.3±1.2	65.9±1.3	6.3±0.5	0.11	6.5±0.5
6h 150℃	40.9±1.2	6.6±1.3	73.6±1.2	5.0±0.5	0.10	5.1±0.6
12h 150℃	44.5±1.1	6.0±1.6	70.2±1.4	7.0±0.6	0.09	7.1±0.7
6h 180℃	43.0±1.4	4.6±1.6	63.5±1.7	14.5±0.6	0.06	14.6±0.6
12h 180℃	48.9±1.4	3.9±2.0	63.1±1.8	15.1±0.6	0.05	15.1±0.6

* ASL: Acid soluble lignin; AIL: Acid insoluble lignin

Table S2 Yield and composition of lignin under different conditions

Conditions	Lignin(%)	
	Yield	Purity
control	/	/
6h 90℃	9.3±0.8	62.8±2.5
12h 90℃	15.3±1.0	72.2±2.0
6h 120℃	53.2±1.0	86.5±1.8
12h 120℃	71.6±1.1	90.0±1.8
6h 150℃	81.5±1.1	91.3±1.8
12h 150℃	47.8±1.0	68.4±1.9
6h 180℃	25.5±1.1	45.6±1.9
12h 180℃	19.±1.2	38.8±2.0
